# Extracting spatial networks from capture–recapture data reveals individual site fidelity patterns within a marine mammal’s spatial range

**DOI:** 10.1002/ece3.8616

**Published:** 2022-02-18

**Authors:** Tyler R. Bonnell, Robert Michaud, Angélique Dupuch, Véronique Lesage, Clément Chion

**Affiliations:** ^1^ Department of Natural Sciences Université du Québec en Outaouais Gatineau Québec Canada; ^2^ Institut des Sciences de la Forêt Tempérée Université du Québec en Outaouais Ripon Québec Canada; ^3^ 4512 Department of Psychology University of Lethbridge Lethbridge Alberta Canada; ^4^ Groupe de Recherche et d'Éducation sur les Mammifères Marins (GREMM) Québec Québec Canada; ^5^ Fisheries and Oceans Canada Maurice Lamontagne Institute Mont‐Joli Québec Canada

**Keywords:** capture–recapture data, *Delphinapterus leucas*, habitat use, network community detection, photo identification, spatial networks

## Abstract

1. Estimating the impacts of anthropogenic disturbances requires an understanding of the habitat‐use patterns of individuals within a population. This is especially the case when disturbances are localized within a population's spatial range, as variation in habitat use within a population can drastically alter the distribution of impacts.
2. Here, we illustrate the potential for multilevel binomial models to generate spatial networks from capture–recapture data, a common data source used in wildlife studies to monitor population dynamics and habitat use. These spatial networks capture which regions of a population's spatial distribution share similar/dissimilar individual usage patterns, and can be especially useful for detecting structured habitat use within the population's spatial range.
3. Using simulations and 18 years of capture–recapture data from St. Lawrence Estuary (SLE) beluga, we show that this approach can successfully estimate the magnitude of similarities/dissimilarities in individual usage patterns across sectors, and identify sectors that share similar individual usage patterns that differ from other sectors, that is, structured habitat use. In the case of SLE beluga, this method identified multiple clusters of individuals, each preferentially using restricted areas within their summer range of the SLE.
4. Multilevel binomial models can be effective at estimating spatial structure in habitat use within wildlife populations sampled by capture–recapture of individuals, and can be especially useful when sampling effort is not evenly distributed. Our finding of a structured habitat use within the SLE beluga summer range has direct implications for estimating individual exposures to localized stressors, such as underwater noise from shipping or other activities.

## Graphical abstract

We show that multilevel binomial models can be effective at estimating individual site fidelity patterns within wildlife populations sampled by capture–recapture. Applying this approach to an endangered beluga population, we identified multiple clusters of individuals, each preferentially using restricted areas. These results have direct implications for estimating individual exposures to localized stressors, such as underwater noise from shipping.

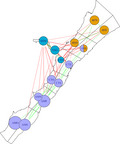

## 1 INTRODUCTION

An understanding of the spatial and temporal distribution of a species of concern is of central importance to conservation and management (Evans & Hammond, 2004). The existence of spatial structuring within populations can have important ecological and management implications. If a population as a whole can be considered as highly mixed, that is, with individuals showing no strong patterns of home range use or substructuring within the wider population, then all individuals are equally likely to feel the impacts of local changes in the environment. In contrast, if the population cannot be considered to be highly mixed, and shows strong substructuring and site fidelity patterns, local stressors might have a disproportionate impact on segments of the population. For example, if noise pollution increased in only one sector, in a highly mixed population all individuals would be lightly impacted, but in a spatially structured population a subset of the population would be highly impacted. These differences in spatial structuring of populations can lead to biased estimation of the likelihood and magnitude of impacts from local stressors both at the individual and population levels (DeFur et al., 2007).

Capture–recapture methods are commonly used to monitor individuals within populations, providing information on vital rates, demography, and insights into within‐population social mixing and habitat use (e.g., Koivuniemi et al., 2016). Photo identification is a long‐recognized method to “capture” individuals with distinct markings (hereafter photo‐ID data) (Urian et al., 2015), and digital photography along with high‐resolution video and machine learning models to identify individuals has led to large capture–recapture datasets (Schneider et al., 2019). Novel statistical and computational methods applied to these capture–recapture datasets have enhanced the potential for quantifying within‐population structures through the use of social network analysis (Perryman et al., 2019; Schilds et al., 2019; Silk et al., 2021).

It is often the case, however, that efforts when collecting capture–recapture data are not evenly distributed. This is especially the case when the population under study occupies a large spatial extent, and where capture methods are not static as in the case with fixed camera traps. This variation in sampling effort can heavily bias estimates of social and spatial networks (Farine & Whitehead, 2015; Hupman et al., 2018; Whitehead, 2008). Datastream permutations have been used to assess potential biases in network estimates from capture–recapture data when estimating networks directly from counts of individuals seen together or in the same regions (Farine, 2017; Silk et al., 2021). Alternatively, state‐space models have been applied to capture–recapture data to include potential sampling biases in estimated networks when based on counts of individuals seen together (Gimenez et al., 2019). Both of these approaches build networks where individuals are the nodes, and the edges represent links between individuals. Here, we propose a multilevel binomial model approach that uses capture–recapture data to estimate spatial networks, that is, where the nodes are spatial regions and the edges between nodes represent the magnitude of similarity in the individual using those regions. By taking this approach, it then becomes possible to quantify spatial structure in habitat use within a population's spatial distribution.

The multilevel binomial modeling approach that we propose to use here does not have a large body of literature to draw on for use with capture–recapture data, but presents unique advantages (Koster & McElreath, 2017). If sampling efforts varies by region within a population's spatial distribution, sighting probability of individuals could be greatly inflated or deflated. The use of a multilevel structure, however, allows for sighting probabilities to be nested per region and expressed in relative terms, that is, as deviations from the mean probability of sighting. This allows the approach to identify the relative magnitude of use of a particular region for each individual. This generates a particular usage profile for each region, that is, which individual highly/lowly use that region (“high users” and “low users” hereafter). It is then possible to quantify how correlated the usage profiles between regions are, providing information about which regions share similar/dissimilar usage profiles. This approach essentially quantifies the extent to which regions share the same individuals while correcting for differences in sampling effort over the course of the study. We suggest that this approach can successfully generate effort‐corrected spatial networks within populations, and can help identify differential patterns in habitat use among individuals and regions.

To evaluate the performance of multilevel binomial models at identifying spatial structuring within animal populations, we first tested the approach with simulated datasets with and without population spatial structuring. We then applied the method to observed data, using a long‐term (18 years) photo‐ID dataset of beluga from the St. Lawrence Estuary (SLE), Canada, and quantified spatial structuring within the population's summer range in the SLE. Finally, we discuss how these estimates of population spatial structuring provide important information for understanding current local stressors and their potential impacts on this endangered and declining population (Lesage, 2021).

## 2 MATERIAL AND METHODS

### 2.1 Study population

The SLE beluga population resides in the lower St. Lawrence during the winter and moves into the upper St. Lawrence in the summer. As this population is endangered and occupies a busy marine traffic area in the summer, there has been considerable effort to understand their distribution and habitat use among other things. In particular, a lot of work has been done to identify hot spots of use and how habitat use varies by age and sex to better mitigate threats to this population (Gosselin et al., 2017; Lefebvre et al., 2012; Lemieux Lefebvre et al., 2018; Michaud, 2005; Ouellet et al., 2021). These studies focused largely on population‐level patterns, such as identifying which areas are used the most. In this study, we use individual‐level data to better understand whether these areas of high use form as a result of nonmobile individuals or of multiple clusters of mobile individuals.

### 2.2 Data

**FIGURE 1:**
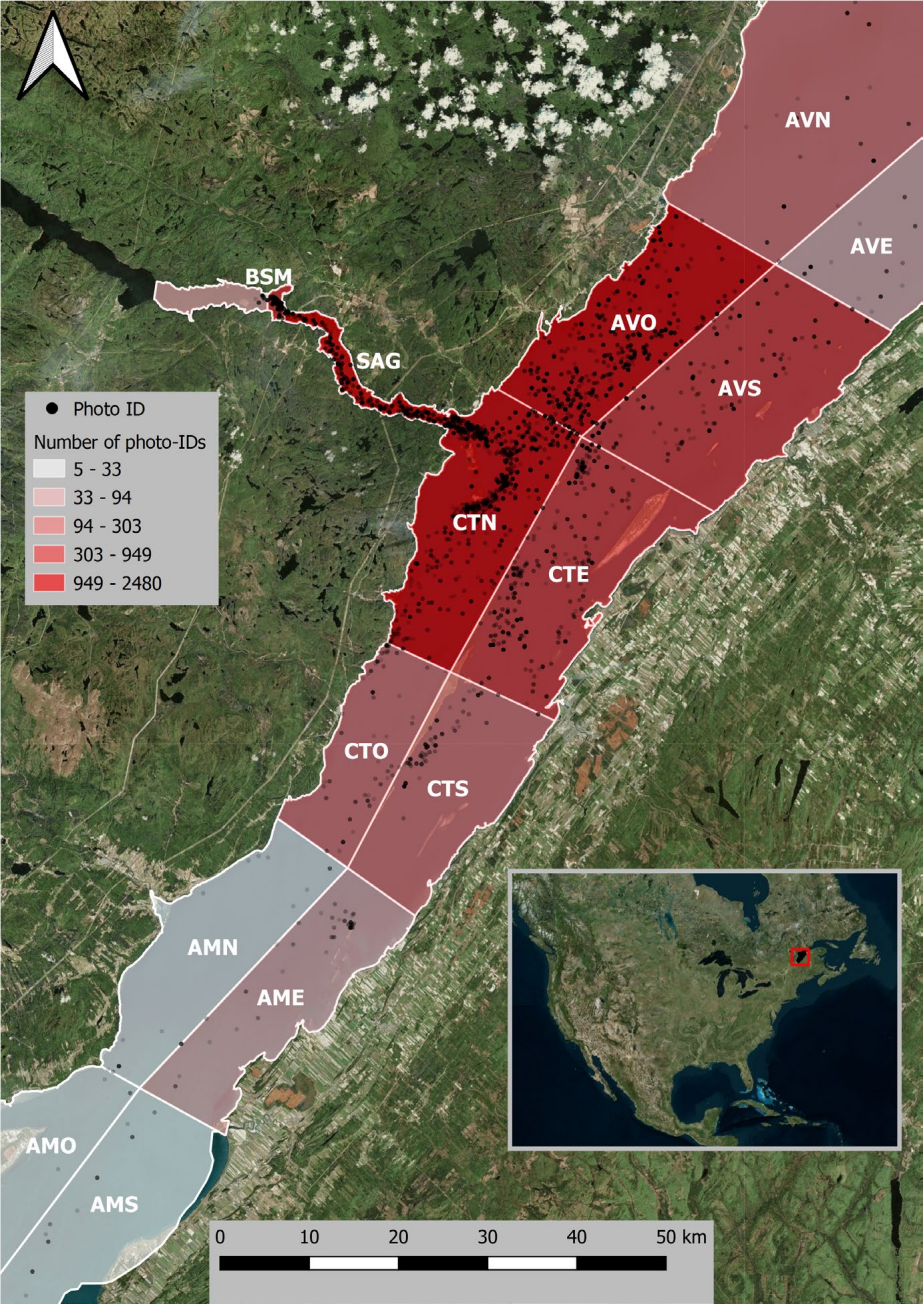
Spatial distribution of each of the 7525 photo identifications (red dots) with the 821 uniquely identified beluga from the St. Lawrence Estuary, Canada (red square in the inset map) over our study period (1989–2007). The 14 sectors are outlined and labeled in white, and cover the summer range of the population

Individual photo‐identification boat surveys were conducted from June to October between 1989 and 2007 as part of an ongoing long‐term study on beluga social organization. The choice of survey area on a given day was selected in a way to avoid resampling areas covered the previous days, and also according to weather conditions. This resulted in approximately 1–4 sectors and 2–5 herds observed on each survey, with only 2% of the individuals captured in two different herds on the same day. When beluga were encountered, the GPS position of the research vessel was noted, and a herd follow was undertaken to photograph as many individuals as possible within the herd using a handheld camera. A herd follow was limited to 3 h, with GPS location noted at least every 30 min. A detailed description of the photo‐ID survey protocol is available in Michaud (2014). Surveys were neither systematic nor random in design, but covered various sectors of a large portion of the population's summer distribution and a broad range of habitats. Sampling effort was unevenly distributed across the summer range divided into three sectors (or stratum), each subdivided into four equal size zones (Figure 1). These sectors were delineated with the marine and middle estuary limits and islands dividing the estuary into south and north channels with respect to prior knowledge of beluga age–sex segregation at the start of the data collection (i.e., 1989).

Each photograph was treated using standard protocols for image selection, scoring, and matching (Urian et al., 2015). Each uniquely identified individual was attributed a resightability index ranging from 1 to 3 based on the degree of distinctiveness of markings. This resulted in a photo‐identification collection of 821 unique individuals that were recaptured on average 9 times over the study period (range 1–90), and which were each associated with a GPS position and sector of initial encounter with the beluga herd. Genetically determined sexes were available for only 29% of the individuals included to the catalog. From individuals with known sex, females had an estimated mean of 12 photo‐IDs and males 17 photo‐IDs, suggesting some bias in terms of capturability (Table S1). Given, age classes were not available for most individuals, and the low percentage of individuals with known sex, the remaining analysis focused on the population as a whole.

### 2.3 Multilevel binomial model

The probability of seeing an individual in each delineated sector of the SLE was estimated using a series of binomial models with correlated random effects. In these models, the dependent variable was the number of times an individual was captured photographically (i.e., photo identified) in a sector. The multilevel structure of the models allowed for the estimation of both the mean probability of photo identifying an individual in each sector and the individual‐level differences in this probability by using individual ID as a random intercept. Furthermore, by allowing for correlation between the individual differences of the sectors it is possible to estimate the similarities in users between the sectors (e.g., if two sectors are highly correlated, this suggests that the individuals in each have the same usage profiles – i.e., individual differences in being seen). If we take, as an example, a case where the study area comprises only two sectors, then the probability of finding individual *i* in a sector can be modeled using a series of multilevel binomial models as:

$$\begin{array}{c} \mathrm{logit}\left(p_{1,i}\right)=\mu_{1}+\;\upsilon_{1i}\\ \mathrm{logit}\left(p_{2,i}\right)=\mu_{2}+\;\upsilon_{2i} \end{array}$$

where $p_{1,i}$ is the probability of seeing beluga i in sector 1, $p_{2,i}$ is the probability of seeing beluga i in sector 2, $\mu_{1}$ and $\mu_{2}$ are the intercepts, that is, the mean probability of seeing a beluga in sectors 1 and 2, and $\upsilon_{1i}$ and $\upsilon_{2i}$ are the estimated individual differences (i.e., random intercepts) from the mean probability of capture in sectors 1 and 2, respectively. The mean probabilities $\mu_{1}$ and $\mu_{2}$ represent preference/avoidance of the specified sector, while $\upsilon_{1i}$ and $\upsilon_{2i}$ are the sector‐specific individual deviations from the mean probability of capture. As a result, it is possible to model the covariance of the individual differences between two sectors using a multivariate normal distribution (Koster & McElreath, 2017):

$$\left[\begin{array}{c} \upsilon_{1i}\\ \upsilon_{2i} \end{array}\right]\;∼\;\mathrm{Multi}\;\mathrm{Normal}\left(0,\;\Omega_{v}\right)$$

$$\Omega_{v}=\left[\begin{array}{cc} \sigma_{1,1} & \sigma_{1,2}\\ \sigma_{2,1} & \sigma_{2,2} \end{array}\right]$$

This multivariate normal distribution has a mean of 0 and a covariance matrix $\Omega_{v}$. Here, the diagonal entries in the covariance matrix ($\sigma_{1,1}$ and $\sigma_{2,2}$) represent the magnitude of individual differences within a sector. This magnitude of individual differences identifies whether there are individual differences in the probability of being seen in a sector (i.e., high values of $\sigma_{1,1}$ and $\sigma_{2,2}$), or whether all individuals are equally likely to be seen (i.e., low values of $\sigma_{1,1}$ and $\sigma_{2,2}$). The off‐diagonal entries ($\sigma_{2,1}$ and $\sigma_{1,2}$) are the covariance estimates between sectors, that is, identifying sectors that share similar user profiles. By converting covariance of individual differences between sectors and correlations, this multilevel modeling approach quantifies how much information individual differences in the probability of being seen in one sector can provide about another sector. Positive correlations suggest that the high/low users in one sector are similarly high/low users in another sector, while negative correlations suggest high/low users in one sector are the low/high users in another sector.

This model can be fit with a Bayesian approach using brms (Bürkner, 2018) with a multilevel syntax: for example, bf(sector 1|trials(*n*) ~ 1 + (1|q|ID)) + bf(sector 2|trials(*n*) ~ 1 + (1|q|ID)) + binomial(). Here, sector 1 indicates how many times each individual was seen in sector 1. While *n* is the total number of times an individual was captured photographically, and allows for the estimation of the probability of being seen when individuals have not all been captured the same number of times. Finally, q represents an arbitrary character choice that allows correlations between the estimates of random intercepts for each sector (Bürkner, 2018).

#### 2.3.1 Dealing with biases in capture–recapture datasets

This multilevel modeling approach accounted for repeated sampling of individuals, and provided an estimate of whether some individuals were seen more or less often than the mean probability of capture in each sector (i.e., $\upsilon_{1i}$ and $\upsilon_{2i}$). Unlike the mean probabilities $\mu_{1}$ and $\mu_{2}$ that represent preference/avoidance of a specified sector, these estimates of individual differences from the mean probability of capture were not impacted by differential sampling among sectors. This was not the case for estimates of the mean probability of capture for each sector (i.e., $\mu_{1}$ and $\mu_{2}$), which were expected to increase in highly sampled sectors. For example, the oversampling of the Saguenay River compared to other sectors (SAG in Figure 1) increased the mean probability of capturing individuals in that sector. However, oversampling of this sector was unlikely to affect the relative probability of being captured among individuals given that all individuals’ chances of being captured were likely to go up or down equally.

Similarly, potential biases due to ease of recognition, for example, some individuals or age classes might bear more distinctive markings than others, are minimized using a multilevel binomial approach as it focuses mainly on differences in the probability of being seen between sectors. For example, if juveniles are 5 times less likely to be successfully photo identified than adults, then they might be less often represented in the photo‐ID database compared to other age classes. However, the difference in distribution of these fewer photo‐identified juveniles across sectors is unlikely to be impacted. For instance, if we successfully photo identified all adults 15 times and all juveniles 3 times, and if both spent twice as much of their time in the Saguenay River compared to all other sectors, then the photo‐ID distribution (seen in vs. outside of the Saguenay River) for adults and juveniles would be expected to be 10:5 and 2:1, respectively. In this example, the capturability varies by age class, but in both age classes the probability of being captured in the Saguenay River would be twice that of the remaining sector. The adaptive partial pooling properties of multilevel models, however, leads individuals with few photo‐IDs, and thus which contain less information, to be less likely to show measurable deviations from the mean probability of being captured. This means that if an age or sex class has very little chance of being identified by photo‐ID (e.g., newborn calves or very young individuals), then they are likely to contribute less to the estimated spatial structures estimated by the multilevel binomial approach.

By using a multilevel modeling approach, we also reduced the chance of false positives when making comparisons between many different individuals in many different sectors (i.e., problem of multiple comparisons). For example, if we were to estimate the differences in the probability of being seen in each sector separately for each individual, the risk of false positives, that is, detecting differences where there is none, would be increased. Instead, if a multilevel approach is used to estimate the differences in probability of being seen it is possible to make effective use of partial pooling to reduce extreme values, especially in cases where the number of recaptures is not equal between individuals. Finally, by running this analysis in a Bayesian framework, we were able to place priors on the individual differences within sectors. In our case, the model was initiated assuming that there were no differences between individuals in their use of each sector, that is, student_t(3,0,1), and as a result no similarity in usage profiles between sectors. These prior choices are particularly useful in sectors with low sampling effort as a form of regularization to avoid overstating conclusions where data are sparse.

### 2.4 Network analysis

Social networks are often used when visualizing and quantifying social structures within populations, with individuals often represented as nodes and their interactions as edges between these nodes (Croft et al., 2008; Farine & Whitehead, 2015). In our case, we used sectors as nodes, and the similarities in user profiles between sectors as edges (i.e., $\sigma_{1,2}$ and $\sigma_{2,1}$). The correlations between sectors estimated from the multilevel binomial model can be used to create a network where the posterior predictions of each correlation parameter correspond to an edge weight in the network. In this way, each edge has a posterior distribution of edge weights and can be used to create many networks from which a distribution of network metrics can be generated. The advantage of having distributions of network measures is that the measures can be readily compared, for example, does one sector have a higher node strength than another? It is also possible to use the distribution of edge weights, and a chosen threshold (e.g., 95% credible interval), to highlight only the edges where the sign of the correlation is known with a particular range of certainty. In this paper, we used this latter approach to generate a signed network (i.e., a network with positive and negative edges). In other words, we created a network where the edges were formed from correlations where the sign was relatively certain, that is, the 95% credible interval does not include zero and was either all positive or negative. We then used a simple signed edge rule to define network communities: where a distinct network community was a set of nodes that shared positive edges but no negative edges. We also made use of signed blockmodeling, an algorithm that can also be used to identify blocks of nodes, and that maximized within‐block positive edges and minimized within‐block negative edges (Doreian & Mrvar, 2015). While the signed edge rule generally provides relatively intuitive results with simple networks, signed blockmodeling is likely to be particularly advantageous when dealing with more complex networks. The network communities detected using these two algorithms were then interpreted as spatial clusters of individuals and not as biological communities.

### 2.5 Testing the modeling approach

The accuracy of the multilevel binomial modeling approach was assessed by generating test datasets from the observed photo‐ID data. We ensured that the test datasets contained the same number of unique individuals, distribution of sightings (i.e., some individuals were seen more than others), and overall number of photo‐IDs as the observed dataset. We, however, varied the spatial location of individual photo‐ID captures in two ways. First, to test if the proposed method correctly detected no pattern when none existed, we created a completely random test dataset by permuting the sector associated with each photo‐ID in the observed dataset. The expected result was to find no correlations between sectors, given that the sectors for each photo‐ID had been randomly permuted. To then test whether the proposed method could also correctly identify patterns when a known pattern existed, we generated a structured test dataset by randomly assigning each uniquely identified individual to four equally populated spatial clusters with the following and hypothetical home range of adjacent sectors: cluster 1 – BSM, SAG, CTN; cluster 2 – CTN, CTO, AMN; cluster 3 – AVO, AVS, AVN; and cluster 4 – AME, CTS, CTE. Following this, we altered the sector of where the individual photo‐IDs were taken so as to fall within sectors associated with an individual's clusters, that is, one of their home range sectors. We did this by choosing a sector for each photo‐ID based on the individual's assigned clusters 80% of the time; a random sector was chosen for the other 20% of the time, introducing noise in the assignment of sectors. We then tested whether the model correctly identified the correlations between sectors that defined the home range of each clusters.

## 3 RESULTS

### 3.1 Testing the modeling approach

**FIGURE 2:**
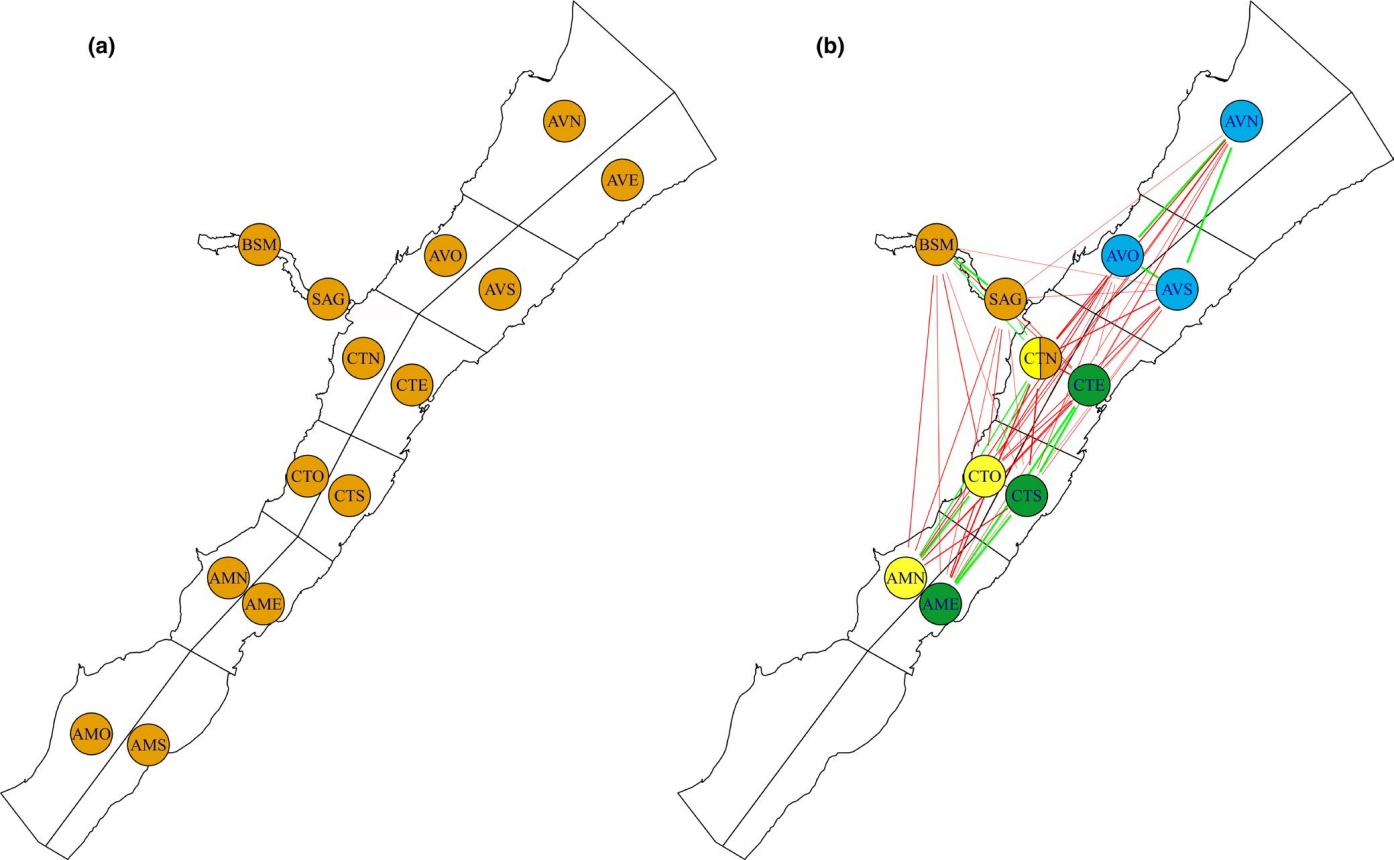
Similarity and dissimilarity between sectors in the simulated datasets: (a) randomly permuted data, where there is no population spatial structure and (b) spatially structured data, where there are four distinct clusters within the population. In (b) the simulated clusters are represented by color codes for each of their sectors (Note: CTN is part of the orange and yellow clusters). The green edges (lines) between two sectors signify that the sectors share high/low users, while red edges (lines) signify that they have opposite high/low users. The lack of an edge signifies that the high/low users of one sector do not provide information about the high/low users of other sectors

When the multilevel binomial model was fit to the data with sectors randomly permuted between all photo‐IDs, the model found as expected no evidence for positive/negative correlations between sectors (Figure 2a), and when we artificially created spatially distinct clusters, the model accurately estimated the correlations between sectors that defined these artificial population spatial structures (Figure 2b). The simple signed edge rule and blockmodeling algorithm applied to the simulated datasets both revealed the four artificially generated spatial clusters, though the blockmodeling algorithm had difficulty with the multimembership node, as it could not assign a node to two blocks (i.e., the CTN node that was shared between clusters 1 and 2).

### 3.2 Quantifying individual variation in habitat use among sectors from observed data

**FIGURE 3:**
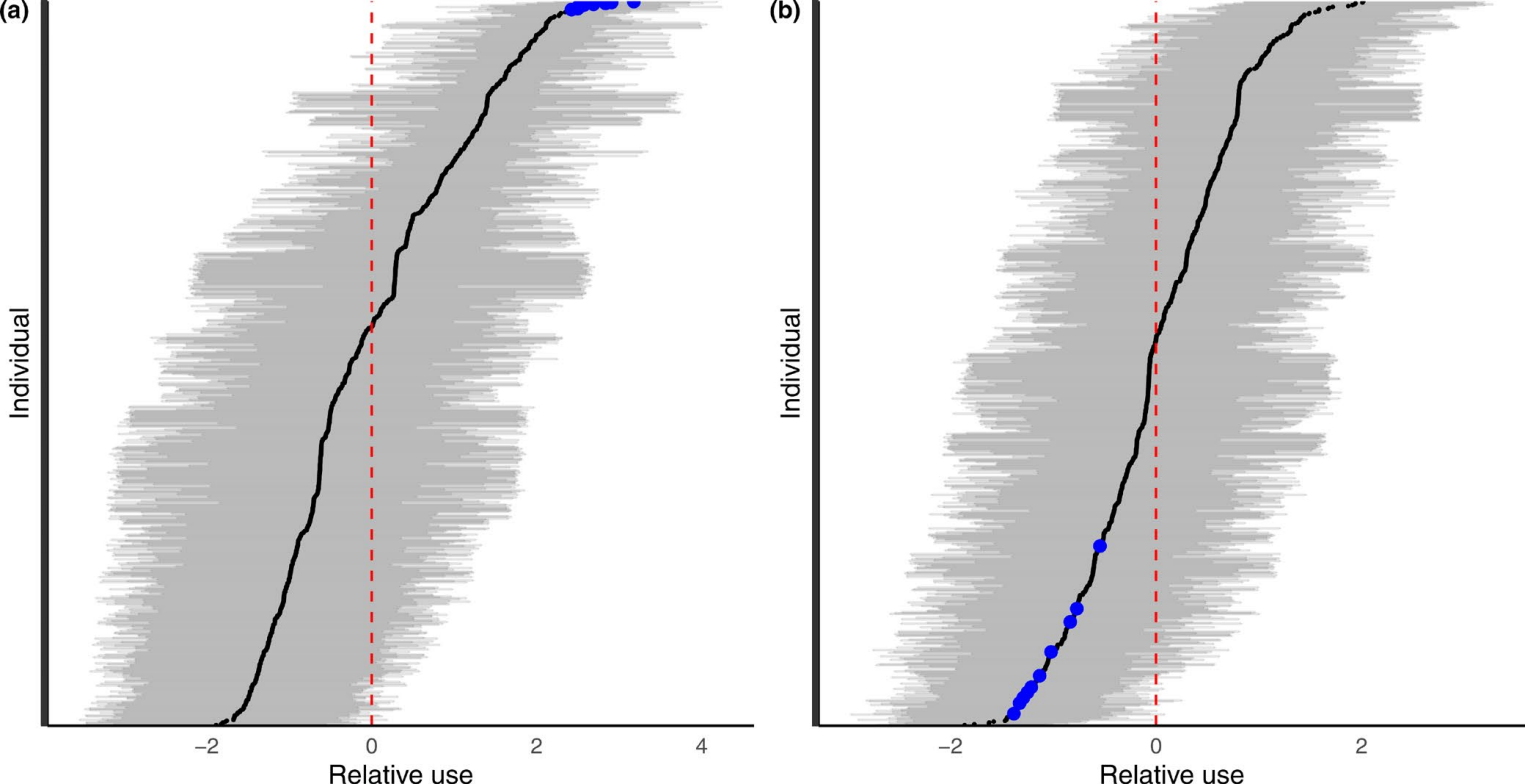
Estimate of the relative use of the (a) SAG and (b) CTE sectors by each photo‐identified individual (i.e., deviation from mean use, $\upsilon_{\mathrm{SAG}i}$ and $\upsilon_{\mathrm{CTE}i}$). The values are deviations (black points) from the mean probability of recapturing individuals within a sector (red dashed line) and are on a logit scale. The horizontal gray lines represent the 95% credible interval. The estimated top 10 users of the SAG sector are represented by blue dots (panel a), and those same individuals are also highlighted in blue in the CTE sector (panel b), illustrating how correlations between sectors were estimated

The model, when applied to the 18 years of observed photo‐ID data, indicated differences between high and low users in all sectors, though the magnitude of these individual differences varied (Table 1). The model also found that these individual differences were correlated between sectors (Table S2), indicating a high magnitude of similarity/dissimilarity between sectors in terms of which beluga used those sectors heavily or rarely. Taking two sectors as examples, for example, the SAG and CTE sectors, the top 10 estimated high users of the SAG (i.e., individuals with a relatively high probability of being found there, blue dots in Figure 3a), were low users of the CTE sector (blue dots in Figure 3b).

**TABLE 1:** Parameter estimates from the multilevel binomial model predicting the probability of capturing an individual by sector

Parameter	Estimate	l−95% CI	u‐95% CI
sd(mu_CTN)	0.6	0.52	0.69
sd(mu_AVS)	0.92	0.78	1.07
sd(mu_CTE)	1.01	0.89	1.14
sd(mu_AMN)	1.15	0.48	1.87
sd(mu_AVO)	1.17	1.05	1.3
sd(mu_CTS)	1.28	1.05	1.53
sd(mu_SAG)	1.41	1.24	1.58
sd(mu_BSM)	1.45	1.23	1.7
sd(mu_CTO)	1.45	1.13	1.8
sd(mu_AVE)	1.48	1.01	2.04
sd(mu_AVN)	1.6	1.19	2.07
sd(mu_AMS)	2.34	1.63	3.21
sd(mu_AME)	2.55	2.04	3.16
sd(mu_AMO)	2.92	1.4	5.34

Notes

Estimated magnitudes of within‐sector individual differences in usage (sd; e.g., $\sigma_{1,1}$) are presented for each sector. Higher estimates indicate higher contrast between high users and low users of that sector, whereas lower estimates indicate a greater homogeneity in usage. To facilitate interpretation, we have ordered the table by lowest to highest estimates of individual differences in usage, and provide the lower and upper 95% credible intervals for each estimate (e.g., l–95% CI, u‐95%CI). As the number of parameters in the model is large, the overall mean by sector (i.e., $\mu_{i}$), and estimated correlations between individual differences (e.g., $\sigma_{2,1}$) are presented in the supplementary section (Table S2).

Our model indicated that the CTN sector was relatively uniformly used by all individuals (i.e., low “sd” value; Table 1) compared to other sectors. In contrast, individual differences in usage were the largest in the AME, AMO, AMS sectors, with some very high/low users of those sectors (Table 1).

### 3.3 Characterizing the population spatial network

**FIGURE 4:**
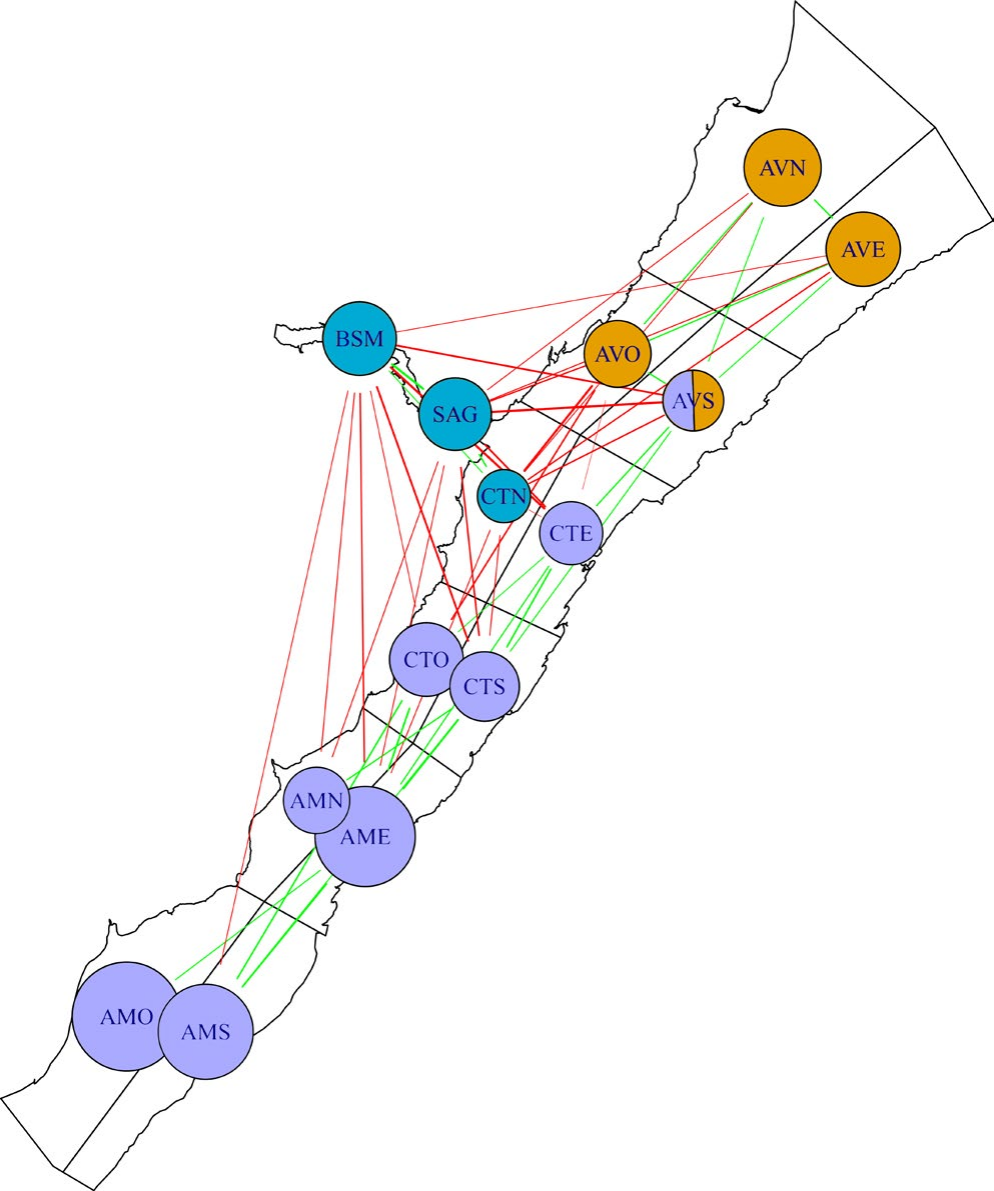
Population spatial structure characterized by similarity and dissimilarity in user profiles between sectors in the SLE beluga population. The green edges between two sectors signify that the sectors share high/low users, while red edges signify that they have opposite high/low users. The lack of an edge signifies that the high/low users of one sector do not provide information about the high/low users of other sectors. Nodes represent sectors, and are colored based the cluster they belong to: that is, shared green edges and no shared red edges. Node sizes represent the magnitudes of individual differences in use within the sector, that is, larger nodes suggest specialized use by a subset of the population

Spatial patterns emerged from using the between sector correlations to generate a signed network overlaid on top of the sectors in the SLE. Applying the simple signed rule and the blockmodeling algorithm to delineate network communities, both indicate that there are three distinct spatial clusters of individuals within the beluga summer range: the lower SLE (AVO, AVS, AVE, AVN), the Saguenay River and mouth (BSM, SAG, CTN), and the upper Estuary and eastern portion of the lower SLE (CTE, CTS, CTO, AME, AMS, AMO, AMN) (Figure 4). In the case of AVS, however, the simple signed rule suggested multimembership for this sector, while the blockmodeling algorithm found AVS to be either: (a) part of the cluster containing (AVO, AVN, AVE) or (b) that the two clusters (orange and purple in Figure 4) merged into one depending on the choice of weighting parameter (i.e., emphasizing positive or negative edges).

## 4 DISCUSSION

Here, we have shown that using capture–recapture data it is possible to estimate spatial networks that can identify spatial structures within populations while controlling unequal sampling effort. Applying this approach to data from beluga in the SLE suggests a non‐random habitat use within the summer range of this population.

In particular, the use of the multilevel binomial model provided information about within‐sector usage patterns of individual beluga. Our results showed that some sectors were predominantly used by a subset of individuals, while other sectors were used more uniformly by all individuals in the population. The CTN sector for instance appeared as a potential high mixing zone for the population, whereas the AME sector seemed to be used by a specific subset of the population. These findings align well with a recent study on habitat connectivity in this population exploiting a different dataset, and suggesting that the CTN sector interconnects strongly with other sectors (Ouellet et al., 2021).

The use of the multilevel binomial model also provided information about the similarity in usage profiles across sectors. Our results across sectors add to the evidence that the beluga population cannot be assumed to be randomly mixing within its summer habitat, suggesting instead the existence of multiple spatial clusters of individuals that make use of particular sectors of the SLE and the Saguenay River. This result suggests, for example, that individuals that are repeatedly seen in the SAG sector are also repeatedly seen in the BSM and CTN sectors, but are seen very little in the CTE and CTS sectors (Figure 4). It should be noted that the identified clusters should not be seen as hard boundaries. For example, we find very little difference between high and low users in the CTN, and that this sector clusters with the BSM and SAG sectors. In this case, the small differences in usage patterns correlated with differences in usage patterns of BSM and SAG. Rather than looking at inclusion within a cluster as a hard boundary, a more nuanced view of a node inclusion within a cluster can be obtained by looking at the amount of individual differences observed within a given sector (i.e., the SD measure Table 1), and the strength of the correlation between sectors (i.e., Table S1).

Our results provide strong evidence that over a period of 18 years, there are regions within the beluga summer range that are being used more often by particular subsections of the population. This suggests that when estimating the impacts of localized stressors on this population, the assumption that individuals are using the Estuary in a similar way will lead to misleading estimates of impact levels. Rather, our results suggest that local stressors are likely to impact certain portions of the population more than others. This unequal distribution of impacts is likely to be particularly exacerbated in cases where exposure to stressors is chronic and cumulative. With the multilevel modeling approach introduced in this paper, it is possible to use capture–recapture datasets to identify if, and to what extent, subsections of the population are using specific areas (Figures 3 and 4). The results from this approach can then be used to help estimate the distribution of impacts within populations as a whole.

A greater understanding of the sex and age segregation in beluga and this population in particular would be beneficial as it would allow spatial networks of these subsections of the population to be estimated, and a better assessment of impacts experienced by these subsections of the population. This is crucial given that juveniles and adult females tend to show less distinctive markings compared to adult males, making captures by photo ID more difficult for these age/sex classes and reducing the amount of information they provide when estimating spatial networks of the population as a whole. Similarly, if the goal is to better estimate the impacts of disturbances within a particular time span, spatial networks can be estimated over shorter time scales (i.e., other than over 18 years used in this study) (e.g., Figures S1 and S2), and can account for the potential of shifts in population habitat use within years (e.g., Figure S5). Both estimating within sub‐subsections of the population and over more targeted time spans will require continued data collection. In this regard, photogrammetric and machine learning work to identify sex, estimate age, and facilitate individual identification is currently underway to refine already collected data and facilitate future data collection.

Properly accounting for animal movements and within‐population site fidelity patterns can lead to drastically different results about impacts of individual stressors or their cumulative effects. For instance, predictions from an agent‐based model of beluga and marine traffic in the STE found that if beluga spend more time within the Saguenay River Sector they likely experience reduced exposure to noise pollution (Chion et al., 2021). This suggests that subsets of individuals within the larger population that use this sector will have reduced noise exposure. This refuge effect, however, is predicted to be lost under scenarios where additional marine traffic is added to the Saguenay River (Chion et al., 2021).

Finally, when implementing multilevel binomial models on other capture–recapture datasets, the use of test datasets should hold a prominent role in the analysis. The use of permutation/simulation methods to both generate spatially structured and unstructured datasets, while maintaining the sample size distribution of the original datasets, can be very valuable in helping to set model priors and to interpret the final model results. The use of permutation approaches is common in social network analysis (Croft et al., 2011; Farine, 2017), and similarly, the use of simulated datasets is becoming more common in statistical workflows more generally (Gelman et al., 2013; McElreath, 2020).

## 5 CONCLUSIONS

We have introduced the use of multilevel binomial models to estimate spatial networks from a capture–recapture approach that is gaining in applicability, that is, photo‐ID data. We have shown, using test datasets, that the proposed method is effective at detecting population spatial structures – quantifying the extent to which subsections of the population make use of specific regions of the populations spatial range. When applied to 18 years of photo‐ID data from an endangered population of beluga in the SLE, our results provide evidence that the population is composed of multiple spatial clusters of individuals with distinct habitat‐use patterns. We suggest that the ability to estimate habitat‐use patterns within animal populations monitored by capture–recapture sampling will contribute to better impact assessments with direct implications for conservation and management.

## AUTHOR CONTRIBUTION

**Tyler R. Bonnell:** Conceptualization (lead); Formal analysis (lead); Methodology (lead); Writing – original draft (equal); Writing – review & editing (equal). **Robert Michaud:** Conceptualization (equal); Data curation (lead); Funding acquisition (equal); Project administration (equal); Resources (equal); Writing – review & editing (equal). **Angélique Dupuch:** Conceptualization (equal); Methodology (equal); Writing – review & editing (equal). **Véronique Lesage:** Conceptualization (equal); Methodology (equal); Writing – review & editing (equal). **Clément Chion:** Conceptualization (equal); Funding acquisition (equal); Methodology (equal); Project administration (equal); Writing – review & editing (equal).

## Supporting information

Supplementary Material

## Data Availability

The permuted and simulated photo‐ID datasets are available on github (https://github.com/tbonne/photoID_multilevel_binomial), along with code used in the analysis.
