# Supplementary material for: Extracting spatial networks from capture–recapture data reveals individual site fidelity patterns within a marine mammal’s spatial range
Source: Ecol Evol. 2022 Feb 18;12(2):e8616. doi: 10.1002/ece3.8616 (PMC8855333; doi:10.1002/ece3.8616)
Supplement: Supplementary file 1 — Supplementary Material [file ECE3-12-e8616-s001.docx]

**Supplementary material:**

*Sex biases in captures:*

To estimate the extent to which males and females differ in their rates of capture we fit a poission regression to estimate mean rates of capture for males and females. The model results showed overdispersion and so was run with observation level random effects, which alleviated the overdispersion (Harrison, 2014). The model results suggest that males have a higher capturability (Table 1), with an estimated mean number of captures of 17 (95%CI: 15, 19) for males and 12 (95%CI: 10, 15) for females.

Table S1: Parameter estimates from a model estimating the number of photo identification captures by sex. The reference sex category is ‘unknown.’

| **Parameter** | **Estimate** | **SD** | **l-95% CI** | **u-95% CI** |
| --- | --- | --- | --- | --- |
| Intercept | 1.28 | 0.04 | 1.20 | 1.35 |
| Sexe (Female) | 1.21 | 0.11 | 1.00 | 1.41 |
| Sexe (Male) | 1.56 | 0.07 | 1.42 | 1.70 |
| sd(Intercept) | 0.73 | 0.02 | 0.68 | 0.78 |

*Full model table:*

Table S2: Parameter estimates from the multilevel multinomial model predicting probability of capturing a photo-ID by sector. Estimated mean probability (µ_i_, logit scale), magnitude of individual differences in mean probability (e.g.,$\upsilon_{1i}$ σ_i,i_), and correlation of individual differences between the different sectors (e.g., σ_i,j_) are presented along with an estimate of their 95% credible intervals. Positive correlations suggest that the high/low users in one sector are similarly high/low users in another sector, while negative correlations suggest high/low users in one sector are the low/high users in another sector.

| **Type** | **Parameter** | **Estimate** | **l-95% CI** | **u-95% CI** |
| --- | --- | --- | --- | --- |
| **Mean probability** µ_i_ **(logit scale)** | |  |  |  |
|  | mu_AME | -5.85 | -6.43 | -5.33 |
|  | mu_AMN | -5.60 | -6.15 | -5.17 |
|  | mu_AMO | -6.68 | -7.41 | -6.08 |
|  | mu_AMS | -6.33 | -7.00 | -5.73 |
|  | mu_AVE | -5.47 | -5.99 | -5.02 |
|  | mu_AVN | -5.06 | -5.52 | -4.65 |
|  | mu_AVO | -1.82 | -1.93 | -1.72 |
|  | mu_AVS | -2.90 | -3.04 | -2.76 |
|  | mu_BSM | -3.78 | -4.04 | -3.52 |
|  | mu_CTE | -2.20 | -2.30 | -2.10 |
|  | mu_CTN | -1.24 | -1.30 | -1.18 |
|  | mu_CTO | -4.58 | -4.92 | -4.28 |
|  | mu_CTS | -4.09 | -4.35 | -3.85 |
|  | mu_SAG | -2.78 | -2.96 | -2.61 |
| **Individual Differences (σ_i,i_**) | |  |  |  |
|  | sd(mu_AME) | 2.00 | 1.64 | 2.38 |
|  | sd(mu_AMN) | 0.76 | 0.14 | 1.31 |
|  | sd(mu_AMO) | 0.65 | 0.05 | 1.41 |
|  | sd(mu_AMS) | 1.53 | 1.10 | 1.98 |
|  | sd(mu_AVE) | 1.16 | 0.78 | 1.57 |
|  | sd(mu_AVN) | 1.30 | 0.98 | 1.65 |
|  | sd(mu_AVO) | 0.78 | 0.68 | 0.88 |
|  | sd(mu_AVS) | 0.83 | 0.70 | 0.97 |
|  | sd(mu_BSM) | 1.30 | 1.09 | 1.52 |
|  | sd(mu_CTE) | 0.78 | 0.68 | 0.89 |
|  | sd(mu_CTN) | 0.33 | 0.27 | 0.39 |
|  | sd(mu_CTO) | 1.27 | 1.00 | 1.55 |
|  | sd(mu_CTS) | 1.18 | 0.97 | 1.40 |
|  | sd(mu_SAG) | 1.16 | 1.01 | 1.31 |
| **Correlations between individual differences (σ_i,j_**) | | |  |  |
|  | cor(mu_AME,mu_AMN) | 0.39 | -0.03 | 0.71 |
|  | cor(mu_AME,mu_AMO) | 0.27 | -0.26 | 0.67 |
|  | cor(mu_AMN,mu_AMO) | 0.14 | -0.36 | 0.57 |
|  | cor(mu_AME,mu_AMS) | 0.63 | 0.39 | 0.82 |
|  | cor(mu_AMN,mu_AMS) | 0.25 | -0.19 | 0.62 |
|  | cor(mu_AMO,mu_AMS) | 0.27 | -0.26 | 0.67 |
|  | cor(mu_AME,mu_AVE) | 0.18 | -0.14 | 0.46 |
|  | cor(mu_AMN,mu_AVE) | 0.15 | -0.28 | 0.56 |
|  | cor(mu_AMO,mu_AVE) | 0.10 | -0.36 | 0.52 |
|  | cor(mu_AMS,mu_AVE) | 0.05 | -0.32 | 0.41 |
|  | cor(mu_AME,mu_AVN) | -0.04 | -0.33 | 0.25 |
|  | cor(mu_AMN,mu_AVN) | -0.02 | -0.42 | 0.39 |
|  | cor(mu_AMO,mu_AVN) | 0.00 | -0.42 | 0.44 |
|  | cor(mu_AMS,mu_AVN) | -0.07 | -0.41 | 0.28 |
|  | cor(mu_AVE,mu_AVN) | 0.50 | 0.19 | 0.75 |
|  | cor(mu_AME,mu_AVO) | -0.21 | -0.40 | 0.00 |
|  | cor(mu_AMN,mu_AVO) | 0.03 | -0.35 | 0.40 |
|  | cor(mu_AMO,mu_AVO) | -0.06 | -0.45 | 0.36 |
|  | cor(mu_AMS,mu_AVO) | -0.28 | -0.54 | 0.01 |
|  | cor(mu_AVE,mu_AVO) | 0.48 | 0.22 | 0.71 |
|  | cor(mu_AVN,mu_AVO) | 0.50 | 0.28 | 0.69 |
|  | cor(mu_AME,mu_AVS) | 0.13 | -0.10 | 0.36 |
|  | cor(mu_AMN,mu_AVS) | 0.21 | -0.16 | 0.53 |
|  | cor(mu_AMO,mu_AVS) | 0.13 | -0.31 | 0.50 |
|  | cor(mu_AMS,mu_AVS) | 0.03 | -0.26 | 0.32 |
|  | cor(mu_AVE,mu_AVS) | 0.40 | 0.12 | 0.66 |
|  | cor(mu_AVN,mu_AVS) | 0.38 | 0.14 | 0.60 |
|  | cor(mu_AVO,mu_AVS) | 0.54 | 0.38 | 0.69 |
|  | cor(mu_AME,mu_BSM) | -0.57 | -0.75 | -0.38 |
|  | cor(mu_AMN,mu_BSM) | -0.37 | -0.66 | 0.00 |
|  | cor(mu_AMO,mu_BSM) | -0.22 | -0.62 | 0.26 |
|  | cor(mu_AMS,mu_BSM) | -0.37 | -0.62 | -0.10 |
|  | cor(mu_AVE,mu_BSM) | -0.34 | -0.59 | -0.06 |
|  | cor(mu_AVN,mu_BSM) | -0.22 | -0.44 | 0.02 |
|  | cor(mu_AVO,mu_BSM) | -0.16 | -0.32 | 0.01 |
|  | cor(mu_AVS,mu_BSM) | -0.66 | -0.80 | -0.51 |
|  | cor(mu_AME,mu_CTE) | 0.54 | 0.34 | 0.72 |
|  | cor(mu_AMN,mu_CTE) | 0.34 | -0.04 | 0.65 |
|  | cor(mu_AMO,mu_CTE) | 0.21 | -0.29 | 0.60 |
|  | cor(mu_AMS,mu_CTE) | 0.38 | 0.10 | 0.62 |
|  | cor(mu_AVE,mu_CTE) | 0.07 | -0.21 | 0.36 |
|  | cor(mu_AVN,mu_CTE) | -0.11 | -0.35 | 0.14 |
|  | cor(mu_AVO,mu_CTE) | -0.10 | -0.26 | 0.07 |
|  | cor(mu_AVS,mu_CTE) | 0.52 | 0.34 | 0.67 |
|  | cor(mu_BSM,mu_CTE) | -0.80 | -0.89 | -0.68 |
|  | cor(mu_AME,mu_CTN) | -0.26 | -0.49 | -0.03 |
|  | cor(mu_AMN,mu_CTN) | -0.24 | -0.59 | 0.16 |
|  | cor(mu_AMO,mu_CTN) | -0.12 | -0.50 | 0.32 |
|  | cor(mu_AMS,mu_CTN) | -0.09 | -0.39 | 0.21 |
|  | cor(mu_AVE,mu_CTN) | -0.53 | -0.77 | -0.26 |
|  | cor(mu_AVN,mu_CTN) | -0.44 | -0.67 | -0.18 |
|  | cor(mu_AVO,mu_CTN) | -0.75 | -0.86 | -0.60 |
|  | cor(mu_AVS,mu_CTN) | -0.63 | -0.79 | -0.44 |
|  | cor(mu_BSM,mu_CTN) | 0.47 | 0.28 | 0.64 |
|  | cor(mu_CTE,mu_CTN) | -0.25 | -0.46 | -0.05 |
|  | cor(mu_AME,mu_CTO) | 0.63 | 0.43 | 0.79 |
|  | cor(mu_AMN,mu_CTO) | 0.28 | -0.15 | 0.63 |
|  | cor(mu_AMO,mu_CTO) | 0.16 | -0.30 | 0.56 |
|  | cor(mu_AMS,mu_CTO) | 0.55 | 0.27 | 0.78 |
|  | cor(mu_AVE,mu_CTO) | -0.12 | -0.45 | 0.22 |
|  | cor(mu_AVN,mu_CTO) | -0.24 | -0.54 | 0.07 |
|  | cor(mu_AVO,mu_CTO) | -0.55 | -0.72 | -0.36 |
|  | cor(mu_AVS,mu_CTO) | -0.16 | -0.39 | 0.07 |
|  | cor(mu_BSM,mu_CTO) | -0.33 | -0.53 | -0.12 |
|  | cor(mu_CTE,mu_CTO) | 0.39 | 0.17 | 0.60 |
|  | cor(mu_CTN,mu_CTO) | 0.19 | -0.06 | 0.42 |
|  | cor(mu_AME,mu_CTS) | 0.70 | 0.53 | 0.85 |
|  | cor(mu_AMN,mu_CTS) | 0.42 | 0.00 | 0.73 |
|  | cor(mu_AMO,mu_CTS) | 0.21 | -0.27 | 0.61 |
|  | cor(mu_AMS,mu_CTS) | 0.42 | 0.14 | 0.67 |
|  | cor(mu_AVE,mu_CTS) | 0.16 | -0.15 | 0.45 |
|  | cor(mu_AVN,mu_CTS) | 0.06 | -0.23 | 0.33 |
|  | cor(mu_AVO,mu_CTS) | -0.04 | -0.23 | 0.15 |
|  | cor(mu_AVS,mu_CTS) | 0.42 | 0.20 | 0.61 |
|  | cor(mu_BSM,mu_CTS) | -0.72 | -0.85 | -0.55 |
|  | cor(mu_CTE,mu_CTS) | 0.64 | 0.46 | 0.80 |
|  | cor(mu_CTN,mu_CTS) | -0.37 | -0.57 | -0.14 |
|  | cor(mu_CTO,mu_CTS) | 0.48 | 0.25 | 0.68 |
|  | cor(mu_AME,mu_SAG) | -0.43 | -0.61 | -0.24 |
|  | cor(mu_AMN,mu_SAG) | -0.36 | -0.66 | 0.00 |
|  | cor(mu_AMO,mu_SAG) | -0.17 | -0.56 | 0.29 |
|  | cor(mu_AMS,mu_SAG) | -0.22 | -0.47 | 0.03 |
|  | cor(mu_AVE,mu_SAG) | -0.44 | -0.68 | -0.19 |
|  | cor(mu_AVN,mu_SAG) | -0.37 | -0.57 | -0.16 |
|  | cor(mu_AVO,mu_SAG) | -0.48 | -0.60 | -0.35 |
|  | cor(mu_AVS,mu_SAG) | -0.80 | -0.90 | -0.68 |
|  | cor(mu_BSM,mu_SAG) | 0.83 | 0.74 | 0.91 |
|  | cor(mu_CTE,mu_SAG) | -0.68 | -0.79 | -0.55 |
|  | cor(mu_CTN,mu_SAG) | 0.67 | 0.52 | 0.80 |
|  | cor(mu_CTO,mu_SAG) | -0.12 | -0.32 | 0.09 |
|  | cor(mu_CTS,mu_SAG) | -0.62 | -0.77 | -0.45 |

*Model run using different aggregation choices*

In this section we rerun our model using different scale choices. Our initial model estimated the spatial structuring of the entire population over 18 years. This provided an estimate that reflects the extent to which a subset of the individuals in the population used specific sectors, and delineates variation in habitat use over this time period. This scale choice is used to assess if there is evidence that some individuals would be more impacted by localized disturbances than others over these 18 years? We now run a series of models to elaborate on this pattern, to assess the same question of how these patterns provide evidence for subsections of the population experiencing more impacts.

We first investigate the temporal stability of the pattern of spatial structure and look at separate 8 year periods. When we run the model on the years 1989-1998 (n=4,655) we find very similar patterns to the entire 18 year period. However, when we run the model on the years 1999-2007 (n=2,780) we find less evidence of a spatial patterns in habitat use. Given the model starts off assuming no similarities/dissimilarities in users between sectors, i.e., priors centered on zero, the change in pattern between 1989-1998 and 1999-2007 can in part be explained by a reduction in data. Similarly, as the few patterns found in the 1999-2007 period match with edges in the 1989-1999 time period, we do not have strong evidence for a changing pattern between these two time periods.


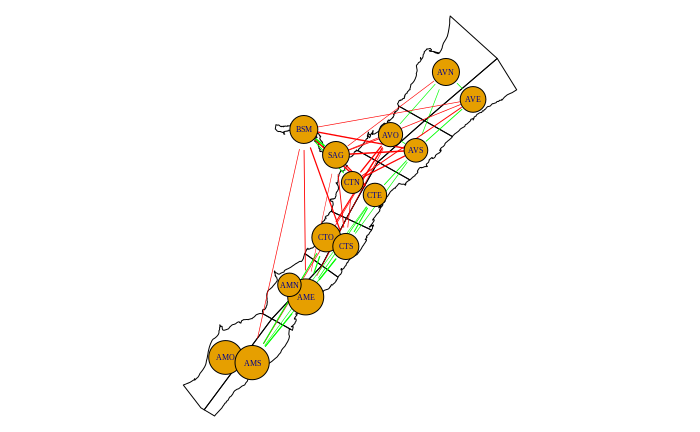


Figure S1: Population spatial structure characterized by similarity and dissimilarity in user profiles between sectors in the St. Lawrence Estuary beluga population for the years 1989-1999. The green edges between two sectors signify that the sectors share high/low users, while red edges signify that they have opposite high/low users. The lack of an edge signifies that the high/low users of one sector does not provide information about the high/low users of other sectors. Nodes represent sectors, and are coloured based the cluster they belong to: i.e., shared green edges, and no shared red edges. Node sizes represent the magnitudes of individual differences in use within the sector, i.e., larger nodes suggest specialized use by a subset of the population.
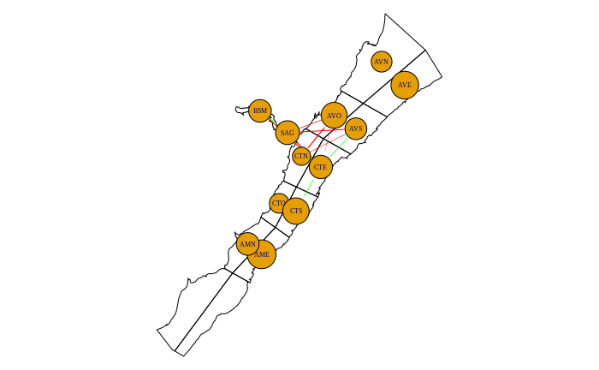


Figure S2: Population spatial structure characterized by similarity and dissimilarity in user profiles between sectors in the St. Lawrence Estuary beluga population for the years 1999-2007. The green edges between two sectors signify that the sectors share high/low users, while red edges signify that they have opposite high/low users. The lack of an edge signifies that the high/low users of one sector does not provide information about the high/low users of other sectors. Nodes represent sectors, and are coloured based the cluster they belong to: i.e., shared green edges, and no shared red edges. Node sizes represent the magnitudes of individual differences in use within the sector, i.e., larger nodes suggest specialized use by a subset of the population.

When we then split the data to look for spatial patterns within sex categories to ask the question: do we have evidence that subsections of the female/male population are more likely impacted than others? When we run the model on females only (1989-2007, n=864) we find only patterns of dissimilarity.


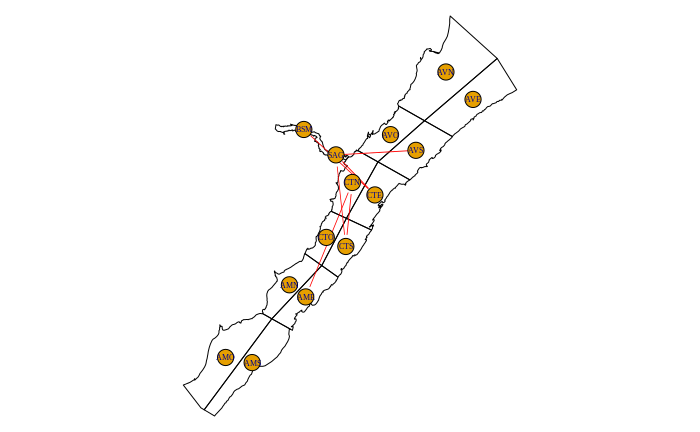


Figure S3: Population spatial structure characterized by similarity and dissimilarity in female user profiles between sectors in the St. Lawrence Estuary beluga population for the years 1989-2007. The green edges between two sectors signify that the sectors share high/low users, while red edges signify that they have opposite high/low users. The lack of an edge signifies that the high/low users of one sector does not provide information about the high/low users of other sectors. Nodes represent sectors, and are coloured based the cluster they belong to: i.e., shared green edges, and no shared red edges. Node sizes represent the magnitudes of individual differences in use within the sector, i.e., larger nodes suggest specialized use by a subset of the population.

While when we run the model with the males only (1989-2007, n = 2,634) we find both similarities and dissimilarities, and those found match the population level estimates.


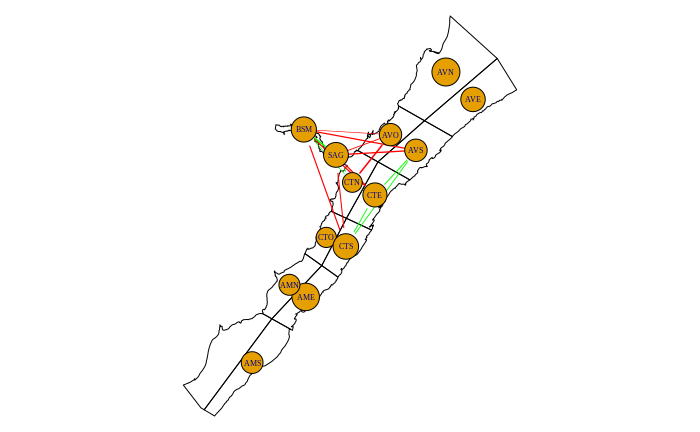


Figure S4: Population spatial structure characterized by similarity and dissimilarity in male user profiles between sectors in the St. Lawrence Estuary beluga population for the years 1989-2007. The green edges between two sectors signify that the sectors share high/low users, while red edges signify that they have opposite high/low users. The lack of an edge signifies that the high/low users of one sector does not provide information about the high/low users of other sectors. Nodes represent sectors, and are coloured based the cluster they belong to: i.e., shared green edges, and no shared red edges. Node sizes represent the magnitudes of individual differences in use within the sector, i.e., larger nodes suggest specialized use by a subset of the population.

Similar to the results of comparing between time periods, we see a reduction in the amount of structure found when comparing male and female only subsections of the beluga population. This reductions is likely due in part to the reduction in data. Similarly, the edges that are found in these male/female only populations are present in the model run on the full dataset again suggesting we do not have strong evidence of a change in structure when looking at these subsections of the population.

*Model run accounting for yearly changes in habitat use by the population*

In this section we re-run the model with a random intercept for year of data collection. The idea here is that if the population is shifting its habitat use from year to year this model will be able to pick that up. Similarly, by accounting for this possible yearly shifting in habitat use, we can better estimate individual habitat use with the random intercept for ID. When this model is run we found very similar patterns to the model where only a random intercept for ID was used (Fig. S4). This suggests that the habitat use patterns capture are not the result of ephemeral movement patterns of individual beluga within a year, but rather consistent patterns of habitat use across years.


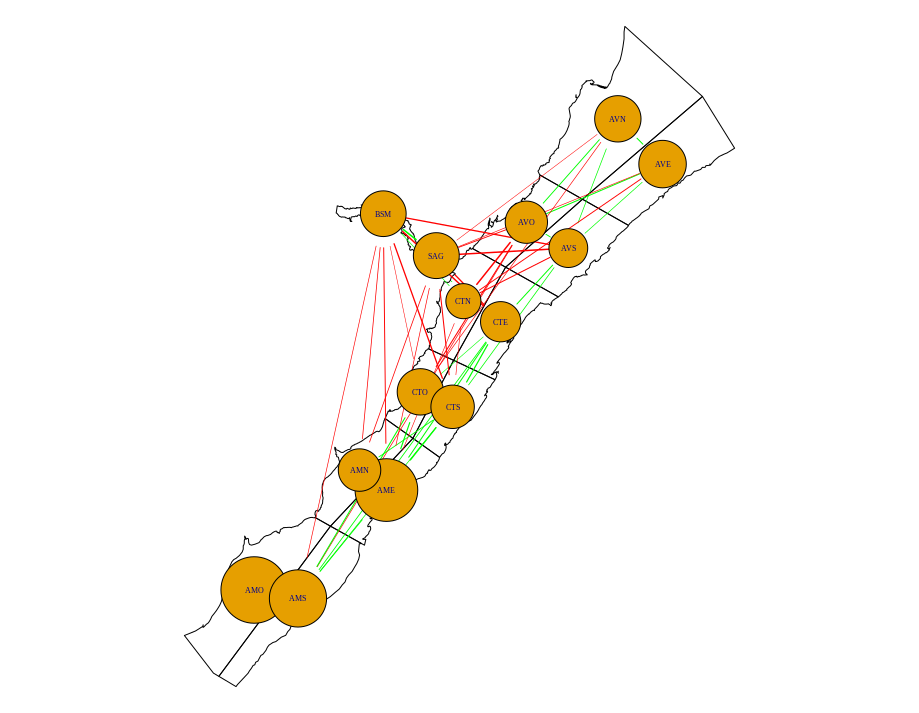


Figure S5: Population spatial structure characterized by similarity and dissimilarity in user profiles between sectors in the St. Lawrence Estuary beluga population accounting for potential within year population habitat use changes. The green edges between two sectors signify that the sectors share high/low users, while red edges signify that they have opposite high/low users. The lack of an edge signifies that the high/low users of one sector does not provide information about the high/low users of other sectors. Nodes represent sectors, and are coloured based the cluster they belong to: i.e., shared green edges, and no shared red edges. Node sizes represent the magnitudes of individual differences in use within the sector, i.e., larger nodes suggest specialized use by a subset of the population.

*References:*

Harrison, X. A. (2014). Using observation-level random effects to model overdispersion in count data in ecology and evolution. *PeerJ*, *2*, e616.
